# High-resolution and bias-corrected CMIP5 projections for climate change impact assessments

**DOI:** 10.1038/s41597-019-0343-8

**Published:** 2020-01-20

**Authors:** Carlos Navarro-Racines, Jaime Tarapues, Philip Thornton, Andy Jarvis, Julian Ramirez-Villegas

**Affiliations:** 1 International Center for Tropical Agriculture (CIAT), Cali, Colombia; 2 CGIAR Research Program on Climate Change, Agriculture and Food Security (CCAFS), c/o CIAT, Cali, Colombia; 3 International Livestock Research Institute (ILRI), Nairobi, Kenya; 4 School of Earth and Environment, University of Leeds, Leeds, UK

**Keywords:** Climate-change impacts, Agriculture

## Abstract

Projections of climate change are available at coarse scales (70–400 km). But agricultural and species models typically require finer scale climate data to model climate change impacts. Here, we present a global database of future climates developed by applying the delta method –a method for climate model bias correction. We performed a technical evaluation of the bias-correction method using a ‘perfect sibling’ framework and show that it reduces climate model bias by 50–70%. The data include monthly maximum and minimum temperatures and monthly total precipitation, and a set of bioclimatic indices, and can be used for assessing impacts of climate change on agriculture and biodiversity. The data are publicly available in the World Data Center for Climate (WDCC; cera-www.dkrz.de), as well as in the CCAFS-Climate data portal (http://ccafs-climate.org). The database has been used up to date in more than 350 studies of ecosystem and agricultural impact assessment.

## Abstract (summary)

**Table:** 

Measurement(s)	climate change • precipitation process • precipitation amount • consecutive dry months index per time period • temperature of air
Technology Type(s)	computational modeling technique
Factor Type(s)	spatial region
Sample Characteristic - Environment	climate system
Sample Characteristic - Location	Earth (planet)

Machine-accessible metadata file describing the reported data: 10.6084/m9.figshare.11353664

## Background & Summary

There is a variety of methods to project the impacts of climate change on agriculture and biodiversity. This diversity arises, at least in part, from the difficulty to couple local-scale agricultural or species distribution and abundance models with General Circulation Model (GCM) projections, which are inherently uncertain^1–3^. GCMs can only model earth processes in coarse grid-cells, which are unsuitable for local agricultural studies^4,5^. Most impact models for agriculture and biodiversity require high-resolution environmental data^6,7^.

Some authors (e.g. refs. ^8–10^) argue that original GCM resolutions should be kept so as not to bias or alter the physical plausibility of GCMs. Nevertheless, agricultural and natural landscapes have large spatial variations, particularly in the tropics, where orography, climate (especially precipitation), soils and crop management, vary across small distances^11^. The vast majority of agricultural and biodiversity researchers have used downscaling in impact studies^6,12^ (but see refs. ^13,14^). This is because conservation plans, niche models, crop models, and biodiversity evaluation require high resolution inputs. Downscaling and bias correction of climate model output produces data that allows local rather than regional or global projections of climate change and its impacts^15,16^. Planning, modeling and monitoring can therefore be at municipality, watershed or other sub-national scales^17–21^.

Downscaling techniques range from smoothing and interpolation of GCM anomalies^19^, to statistical modeling, neural networks, and regional dynamical climate modelling^22^. They differ in accuracy, output resolution, computational requirements and climatic science robustness. Dynamical and statistical downscaling are the most frequently used techniques to downscale GCMs for agricultural impact studies^23,24^. Bias-correction, on the other hand, focuses on using different types of statistical techniques to make the climate model output more realistic, and, in many cases (i.e. when observations are available at high spatial resolution), also of greater spatial resolution^15,25^.

Dynamical downscaling uses Regional Climate Models (RCMs) to increase the resolution of climate projections, with boundary and initial conditions from a GCM as inputs^26–28^. RCMs consider more detailed specifications of land use and water bodies, simulate mesoscale processes in more detail than GCMs, and, in some cases, are capable of explicitly resolving convective rainfall processes^29,30^. RCMs are computationally expensive, and require physical understanding of the climate system, time and storage to obtain a single scenario-by-period output^21^. RCM outputs have been made available recently through the Coordinated Regional Climate Downscaling Experiment (CORDEX)^31^. However, given their computational cost, only a handful of RCM–GCM combinations can realistically be used to produce future high-resolution climate change projections^32,33^. Moreover, RCM outputs are also subject to climate model error from both the structure of the RCM and the boundary conditions of the driving GCM^30,34^.

Statistical downscaling (SD) is an easier and computationally less expensive method to develop climate change projections with high spatial resolution^35^. SD typically consists of two steps, (i) developing a statistical relationship between local climate variables and large-scale predictors, and (ii) the application of those statistical models onto future GCM output to derive future downscaled data^36^. SD assumes that climates will only change at coarse scales and that relationships between variables at local scale remain relatively constant in the future period^30,36^. Bias correction (BC) is yet simpler than SD, and is typically implemented by applying a ‘change factor’ or ‘delta’ derived from a GCM onto the historical observations^15,35^. BC can also be implemented by applying a ‘nudging’ factor to the climate model output, or by quantile-mapping of climate model outputs onto observations^16,37^. Since no GCM is a perfect representation of the true climate, BC seeks to correct those attributes in the climate model output that are known or hypothesized to be important for impacts modeling^4,15,38^.

Here, we used BC to develop the CCAFS-Climate global database of bias corrected climate change projections. To develop the database, we applied the delta method (a simple BC) to 35 Coupled Model Intercomparison Project Phase 5 (CMIP5) models^39^, and four representative concentrations pathways (RCPs)^40^. We used the delta method since we focus on providing data for 30-year mean climate conditions, and because the method has already been shown to be robust to correct mean climate conditions in other regions^15^. For each GCM, we used the 30-year future periods named as 2030s (2020–2049), 2050s (2040–2069), 2070s (2060–2089) and 2080s (2070–2099) and three climate variables (mean monthly maximum and minimum temperatures and monthly rainfall). We used the WorldClim global climate database^11^ as the reference set of observations for the historical period. The database is freely available through the World Data Center for Climate (WDCC; cera-www.dkrz.de)^41^, as well as through the CCAFS-Climate data portal (http://ccafs-climate.org). We evaluate the method to quantify the advantages of using bias-corrected climate data in comparison with the original GCM outputs, using a perfect sibling framework (see Methods). Furthermore, we summarize existing applications of the high-resolution gridded datasets produced here in environmental studies characterizations to assess the impacts of climate change on agricultural production, biodiversity, conservation, and water resources. Finally, we discuss the assumptions and limitations in the methods and data.

## Methods

CCAFS-Climate was produced by bias-correcting the original GCM outputs using spatial interpolation of the anomalies or deltas (differences between future and current climates). To this aim, anomalies (‘deltas’) are first calculated using GCM output as the difference between future and historical periods, and then interpolated onto a 30 arc-s grid. We then applied the interpolated anomalies to the baseline climate of the WorldClim high resolution (30 arc-s) surfaces^11^. This method is called delta change or change factor^42,43^ (DC). Our implementation of DC seeks to correct the modeled mean climate from the climate models, which is a critical aspect in understanding crop and species distributions and productivity under climate change^44,45^, while also providing results at high spatial resolution.

### Data acquisition

#### Present-day observed climatology

We used the high spatial resolution (30 arc-s, ~1 km at the Equator) climate datasets of WorldClim^11^. We chose WorldClim due to its high spatial resolution, wide use (i.e. more than 15,000 citations), and quality^11^. WorldClim used data from more of 47,000 weather stations from 1950–2000 worldwide as input to produce interpolations. WorldClim used the thin-plate splines algorithm^46^ to interpolate mean monthly maximum and minimum temperatures, and monthly precipitation. There are other global datasets for both temperature and precipitation^47^, but they use fewer locations or have coarser spatial resolution. Moreover, WorldClim compares well to other global datasets, especially in areas of high weather station density^11,48,49^.

#### General circulation models data

The Coupled Model Intercomparison Project Phase 5 (CMIP5)^39^, coordinated by the World Climate Research Programme in support of the IPCC Fifth Assessment Report (AR5)^50^, provides simulations from state-of-the-art GCMs. CMIP5 provides, for a large number of models, climate projections for all four Representative Concentration Pathways (RCPs)^39^.

We used present day simulations (1961–1990) and future projections (2010–2100) of global climate at original GCM resolution (70–400 Km) from a total of 35 GCMs, and all RCPs, namely, RCP 2.6, 4.5, 6.0 and 8.5 (Table 1)^40^. GCM data included monthly time series of maximum temperature, minimum temperature and precipitation flux. All GCM data were downloaded from the CMIP5 web data portal at https://esgf-node.llnl.gov/projects/cmip5/. Not all GCM-by-RCP combinations were available (see Table 1).

**Table 1:** CMIP5 Global Climate Models.

Model (Reference)	Institute	RCP			
		2.6	4.5	6.0	8.5
BCC-CSM1.1^91–93^	Beijing Climate Center, China Meteorological Administration	O	O	O	O
BCC-CSM1.1(m)^91–93^		O	O	O	O
BNU-ESM^94^	Beijing Normal University	O	O	X	O
CCCMA-CanESM2^95,96^	Canadian Centre for Climate Modelling and Analysis	O	O	X	O
CESM1-BGC^97,98^	National Science Foundation, Department of Energy, National Center for Atmospheric Research	X	O	X	O
CESM1-CAM5^97^		O	O	O	O
CNRM-CM5^99^	Centre National de Recherches Meteorologiques and Centre Europeen de Recherche et Formation Avancees en Calcul Scientifique	O	O	X	O
CSIRO-ACCESS1.0^100,101^	Commonwealth Scientific and Industrial Research Organization (CSIRO) and Bureau of Meteorology (BOM), Australia	X	O	X	O
CSIRO-ACCESS1.3^100,101^		X	O	X	O
CSIRO-Mk3.6.0^102^	Queensland Climate Change Centre of Excellence and Commonwealth Scientific and Industrial Research Organization	O	O	O	O
EC-EARTH^103^	European Centre for Medium-Range Weather Forecasts (ECMWF)	X	X	X	O
FIO-ESM^104^	The First Institute of Oceanography, State Oceanic Administration, China	O	O	O	O
GFDL-CM3^105,106^	NOAA Geophysical Fluid Dynamics Laboratory	O	O	O	O
GFDL-ESM2G^107^		O	O	O	O
GFDL-ESM2M^107^		O	O	O	O
GISS-E2H^108,109^	NASA Goddard Institute for Space Studies USA	O	X	O	O
GISS-E2HCC^108,109^		X	O	X	X
GISS-E2R^108,109^		O	O	O	O
GISS-E2RCC^108,109^		X	O	X	X
INM-CM4^110^	Institute of Numerical Mathematics of the Russian Academy of Sciences	X	O	X	O
IPSL-CM5A-LR^111^	Institut Pierre Simon Laplace	O	O	O	O
IPSL-CM5A-MR^111^		O	O	X	O
IPSL-CM5B-LR^111^		X	X	X	O
LASG-FGOALS-G2^112^	Institute of Atmospheric Physics (LASG) and Tsinghua University (CESS)	O	O	X	O
MIROC-ESM^113^	University of Tokyo, National Institute for Environmental Studies and Japan Agency for Marine-Earth Science and Technology	O	O	O	O
MIROC-ESM-CHEM^113^		O	O	O	O
MIROC-MIROC5^114^		O	O	O	O
MOHC-HadGEM2-CC^115,116^	UK Met Office Hadley Centre	X	O	X	O
MOHC-HadGEM2-ES^115,116^		O	O	O	O
MPI-ESM-LR^117^	Max Planck Institute for Meteorology	O	O	X	O
MPI-ESM-MR^117^		O	X	X	O
MRI-CGCM3^118,119^	Meteorological Research Institute	O	O	O	O
NCAR-CCSM4^120^	US National Centre for Atmospheric Research	O	O	O	O
NCC-NorESM1-M^121^	Norwegian Climate Centre	O	O	O	O
NIMR-HADGEM2-AO^115,116^	National Institute of Meteorological Research and Korea Meteorological Administration	O	O	O	O
Total		26	31	19	33

### Delta method downscaling

**Fig. 1:**
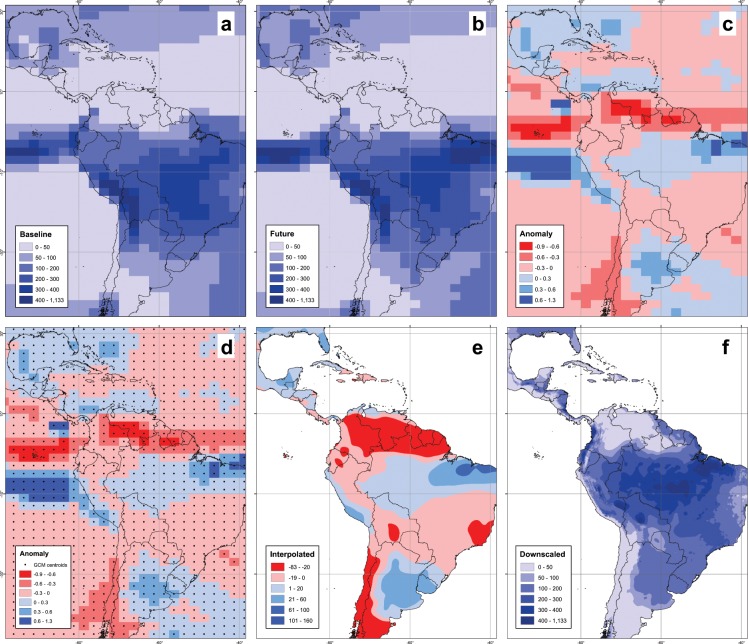
Illustration of the downscaling process with January total precipitation using the GFDL-CM3 GCM pattern. (**a**) Baseline data, (**b**) future data for 2050s (2040–2069 average), (**c**) delta or anomaly by 2050s, (**d**) delta or anomaly by 2050s with GCM centroids (points) overlaid, (**e**) 30 arc-s interpolated anomaly, and (**f**) future downscaled climate surface at 30 arc-second spatial resolution. Values in mm/month.

The DC approach presented here is a simple form of BC in which a change factor or ‘delta’ is derived from the GCM, and then added onto the observations (WorldClim). The purpose of our dataset is to provide a bias-corrected and high-resolution representation of the mean climates, and for this reason we employ the DC approach. The change factor is defined as the difference between the long-term (30-year) mean of a climate variable in the future and the historical period. The method comprises the following steps: (1) calculation of 30-year averages for present-day simulations and 4 future periods; (2) calculation of anomalies as the absolute difference between future and present day values in temperatures (minimum and maximum) and proportional differences in total precipitation; (3) interpolation of these anomalies using centroids of GCM grid cells as points for interpolation; and (4) addition of the interpolated gridded data to the current climates from WorldClim (Fig. 1).

Using the full present-day monthly time-series from the GCM (Sect 0), we thus calculated 30-year means as a baseline (1961–1990), for each GCM and variable. Next, we calculated the 30-year means for each RCP and future period. The future periods are: 2020–2049 (2030s), 2040–2069 (2050s), 2060–2089 (2070s) and 2070–2099 (2080s). For each future period, we calculated the anomaly (delta change) with respect to the baseline climate of the same GCM for each variable and month. We used absolute differences for temperatures (Eq. 1) and relative changes for precipitation (Eq. 2).

1 $$\Delta {X}_{i}={X}_{Fi}-{X}_{Ci}$$

2 $$\Delta {X}_{i}=\frac{{X}_{Fi}-{X}_{Ci}}{{X}_{Ci}}$$

where, *ΔX*_i_ is the delta change, *X*_*Ci*_ the 30-year mean of the variable in the current climate, and *X*_*Fi*_ the 30-year mean of the variable in the future climate of each GCM in the month *i*.

The use of relative changes for precipitation avoids arriving at negative values when applying GCM delta values onto observed WorldClim precipitation. We note that in very dry areas (i.e. monthly historical precipitation close to zero) relative changes could produce unreasonably large relative precipitation increases (e.g. Sahara Desert). To avoid this, we made two adjustments: (1) we set a threshold of 0.1 mm month^−1^ both for current and future GCM values, which prevents indetermination in Eq. 2; and (2) we truncate the top 2% of anomaly values to the 98^th^ percentile value in the empirical probability distribution for each anomaly gridded dataset. Truncation avoids situations in which very low values in the denominator in Eq. 2 lead to very high delta values, which when applied onto WorldClim may lead to unrealistically high values for the future precipitation.

We next apply a thin-plate splines interpolation (TPS)^51,52^ to derive 30 arc-s anomalies. TPS have been used extensively in climate science^11,46,53,54^. The procedure ensures a smooth (continuous and differentiable) surface together with continuous, first-derivative surfaces. Rapid changes in gradient or slope (the first derivative) may occur in the vicinity of the data points. The spline method performs a two-dimensional minimum curvature spline interpolation on a point dataset resulting in a smooth surface that passes exactly through the input points.

Original GCM grid cells are transformed to points with position equal to the centroid of the grid cell, and the TPS interpolation is applied across these points. We used 8 points as neighborhood, though using less (4) or more (12) produced similar results. Interpolations at coastlines are done using relevant ocean grid points from the GCM, only when strictly necessary. The resolution of the resulting interpolation is 30 arc-s, for consistency with WorldClim. This interpolation procedure yields surfaces of changes in climates for each of the 12 months and 3 variables. We produce a total of 36 interpolated surfaces of monthly changes in climates per GCM, RCP and period.

We add anomalies to the baseline climates from WorldClim to get the downscaled future. In the case of temperatures (minimum and maximum temperatures) for each pixel, the anomalies in degree Celsius are simply summed to the actual value in degree Celsius reported in WorldClim (Eq. 3). For precipitation, we use the absolute value of the change relative to the baseline period in order to avoid monthly precipitation values going below 0, and maintain homogeneities with WorldClim (Eq. 4).

3 $${X}_{DCi}={X}_{OBSi}+\Delta {X}_{Ii}$$

4 $${X}_{DCi}={X}_{OBSi}\ast (1+\Delta {X}_{Ii})$$

where, *X*_*OBSi*_ is the current climate from observations (i.e. WorldClim); Δ*X*_*Ii*_ is the interpolated anomaly (delta); and *X*_*DCi*_ is the downscaled future climate of each GCM in the month *i*.

After calculating the corresponding future values for each of the 36 interpolated surfaces, we calculate mean temperatures, assuming a normal distribution in temperatures during the day, as the average of maximum and minimum temperatures.

Beyond the monthly data, we also calculated 19 bioclimatic indices^55,56^ (see full list in Table 2), which have become standard for species distributions modeling for wild and crop species^57,58^. These indices provide descriptions of annual trends (i.e. annual mean temperature, total annual rainfall), seasonality (temperature range, temperature and precipitation standard deviations), and stressful conditions (precipitation during dry or wet periods, temperatures during hot and cold periods).

**Table 2:** List of bioclimatic variables derived from monthly data.

Variable name	Description	Units
bio_1	Annual mean temperature	°C
bio_2	Mean Diurnal Range	°C
bio_3	Isothermality	—
bio_4	Temperature Seasonality	°C
bio_5	Max Temperature of Warmest Month	°C
bio_6	Min Temperature of Coldest Month	°C
bio_7	Temperature Annual Range	°C
bio_8	Mean Temperature of Wettest Quarter	°C
bio_9	Mean Temperature of Driest Quarter	°C
bio_10	Mean Temperature of Warmest Quarter	°C
bio_11	Mean Temperature of Coldest Quarter	°C
bio_12	Total annual precipitation	mm
bio_13	Precipitation of Wettest Month	mm
bio_14	Precipitation of Driest Month	mm
bio_15	Precipitation Seasonality (Coefficient of Variation)	mm
bio_16	Precipitation of Wettest Quarter	mm
bio_17	Precipitation of Driest Quarter	mm
bio_18	Precipitation of Warmest Quarter	mm
bio_19	Precipitation of Coldest Quarter	mm

## Data Records

Our datasets comprise the most comprehensive bias-corrected set of climate change scenarios from IPCC AR5. The DC approach was applied over 35 different GCM outputs (Table 1) from CMIP5^39,50^, four RCPs, and four different 30-year periods, including: 2030s (2020–2049), 2050s (2040–2069), 2060s (2050–2079) and 2080s (2070–2099). The combination of all these settings produce a total of 436 different global scenarios. Each scenario comprises four variables at a monthly time-step: mean, maximum, minimum temperature, and total precipitation, in addition to a set of 19 bioclimatic variables. We provide data at four spatial resolutions, namely, 30 arc-s (~1 km at the Equator), 2.5 arc-min (~5 km), 5 arc-min (~10 km), and 10 arc-min (~20 km). We pack the twelve months of the year for each variable and resolution in Zip archives containing ESRI-Arc/Info binary grids (for 2.5, 5 and 10 arc-min datasets) and ESRI-ASCII grids (all resolutions). Moreover, we offer datasets by tiles (18 tiles in total) to facilitate the data retrieval for users who seek only regional data. All the possible combinations produce 57,552 records which are freely available in a digital table^59^. The complete dataset set is ~7 TB in size. All data are freely available at the World Data Center for Climate (WDCC) repository^41^.

## Technical Validation

**Fig. 2:**
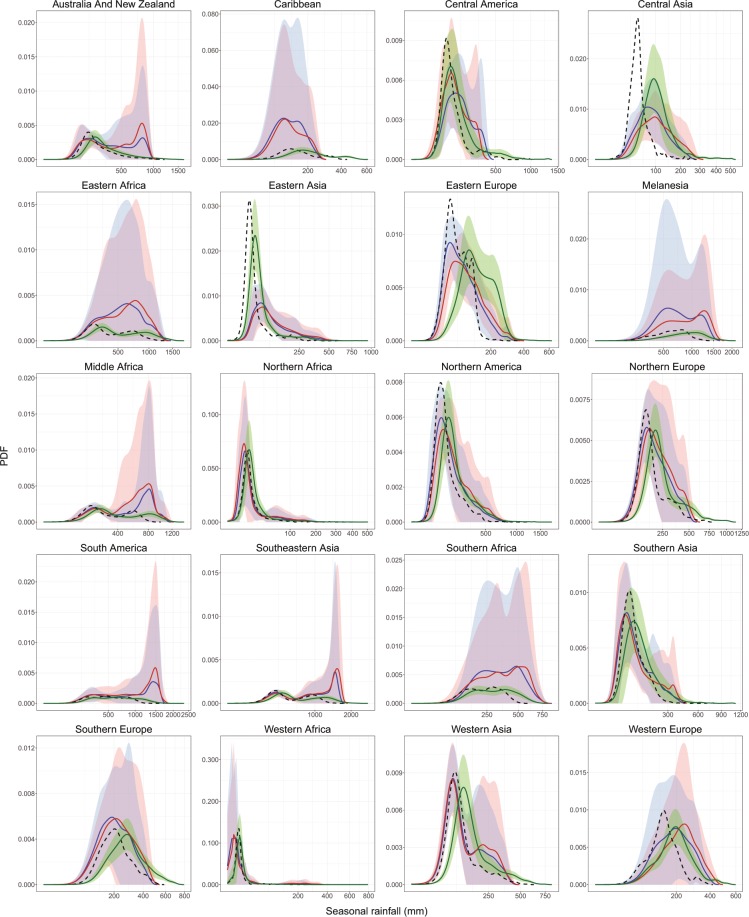
Probability density functions (PDF) of seasonal rainfall for December-January-February season in comparison with observations. The continuous lines belong to PDF average and the shading shows the average ± one standard deviation, for all GCM-future (red), GCM-historical (blue) and DC GCM (green). Dotted line is average PDF for the observations (i.e. WorldClim). The definition of areas of the world follows the United Nations Statistics Division (UNSD)^122^.

The DC strategy focuses on identifying the aspects of the climate model that need correction (in our case the long-term mean), and then uses the observations to find a correction factor for the quantities of interest (e.g. long-term average monthly mean temperature). To illustrate the method, a comparison of the Probability Density Function (PDF) between observations, GCM-historical, GCM-future, and downscaled data is shown in Fig. 2 for precipitation and 3 for temperature, respectively. We chose for this example the highest emission scenario RCP 8.5 which displays greater differences than other scenarios and 2050s –a period of importance for adaptation and policy-making decisions in agriculture both from an adaptation and mitigation perspective, as it is the period around which global mean temperature is projected to exceed 2 °C above pre-industrial levels^60–63^.

The DC approach changes much of the distribution of the mean seasonal temperature and seasonal rainfall in the majority of the world zones studied. In areas such as Australia and New Zealand, South America and Southeast Asia, the DC approach makes both the mean, the variance and the overall PDF distribution more consistent with that of WorldClim. In the Caribbean, Melanesia, Southern Africa, the method appears to correct the systematic underestimation of seasonal rainfall under 500 mm (Fig. 2). These high-frequency and low-intensity events are frequently referred to as the ‘drizzle problem’ in GCMs^34,64,65^. In Central America, GCMs tend to overestimate the distribution of rainfall values under 500 mm but also are not capable of simulating rainfall above 500 mm. In that case, the DC method modifies the variance, bringing it closer to that of WorldClim, although it can introduce some extreme high values that are unlikely to occur.

**Fig. 3:**
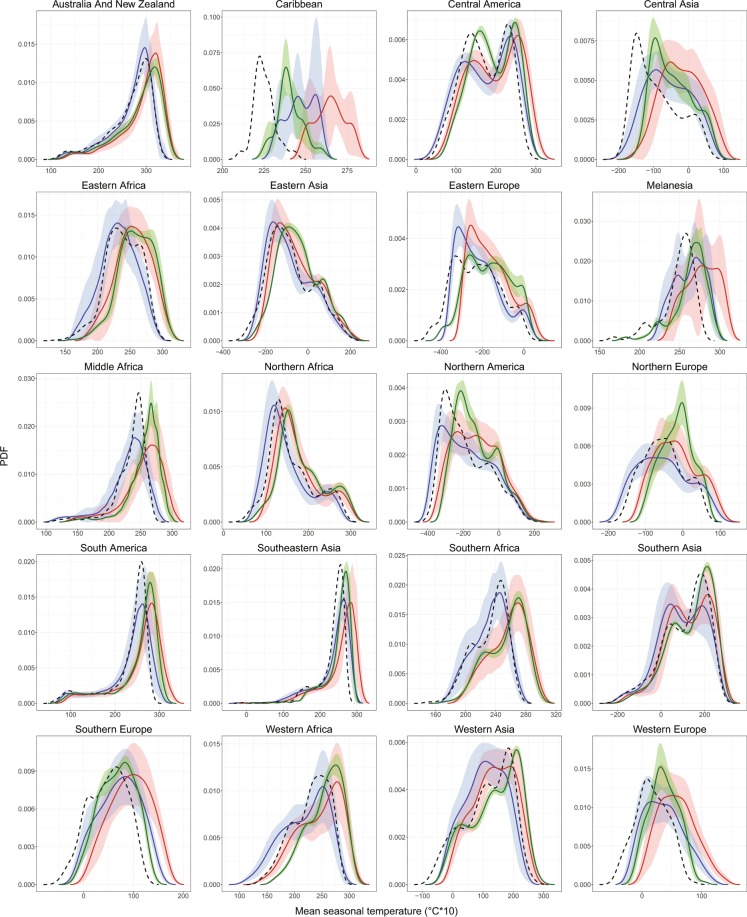
Probability density functions (PDF) of seasonal mean temperature for DJF season in comparison with observations. The continuous lines belongs to PDF average and the shading shows the average ± one standard deviation, for all GCM-future (red), GCM-historical (blue) and DC GCM (green). Dotted line is average PDF for the observations (i.e. WorldClim). The *x*-axis is multiplied by 10 for consistency with the data provided online, which is multiplied by 10 to reduce storage space needs. The definition of areas of the world follows the United Nations Statistics Division (UNSD)^122^.

As for precipitation, historical uncorrected GCM outputs are not capable of representing the PDF of the temperature observations in many cases (Fig. 3). For example, GCMs do not reproduce very low temperatures such as those in Eastern Europe, and low tropical temperatures (Melanesia). The DC approach brings the shape of the PDF of the future projections closer to that of the observed PDF, therefore likely reducing model error. For instance, for temperatures in the Caribbean, the mean of the PDF is changed making so that it is closer to the observations. DC also helps to reproduce very low values (e.g. Eastern and Southern Africa) that are not observable in the original GCM.

**Fig. 4:**
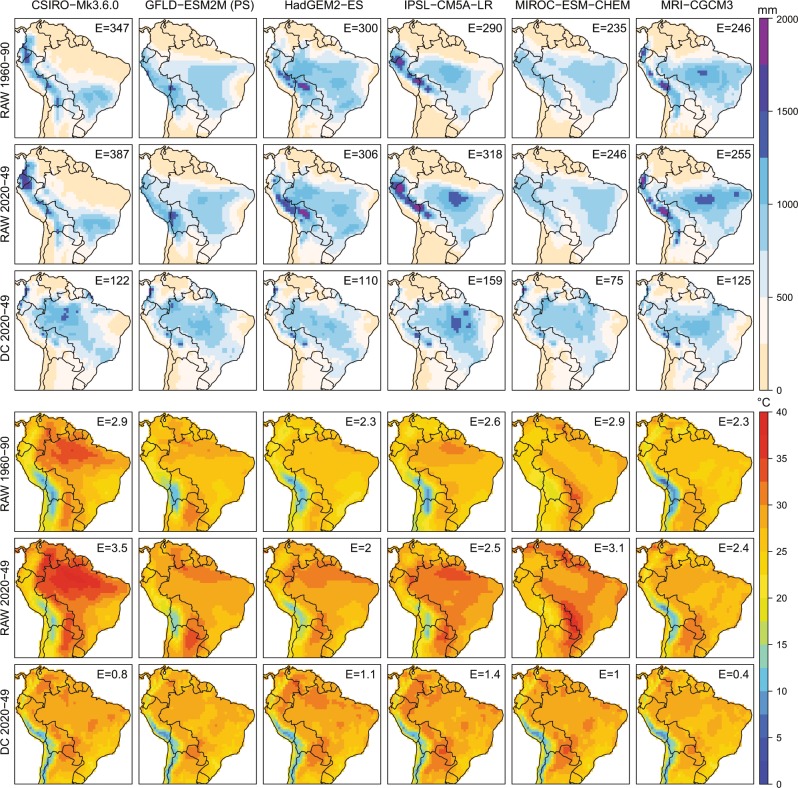
Demonstration of the DC calibration methodology using a range of GCM simulations. Top maps (in blue color scale) show results for DJF seasonal rainfall, and bottom maps (in rainbow color scale) for DJF mean seasonal temperature. GFDL-ESM2M is selected as the “prefect sibling” for verification against the calibrated projections using other GCM data. The RMS error for the region shown is given as the E value in the top-right of the maps.

**Fig. 5:**
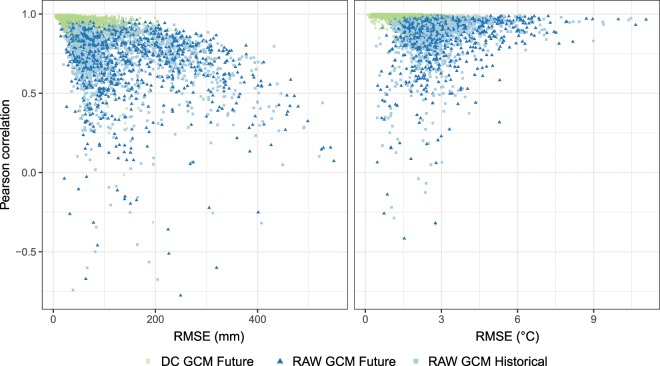
Error evaluation considering all seasons, regions and different combinations of the “Perfect Sibling” model. Left panel shows precipitation, and right panel temperature. Model error, measured using the RMSE and Pearson correlation coefficient are shown for the uncorrected GCMs in the historical period (light blue squares), the uncorrected GCMs in the future (dark blue triangles) and DC calibrated dataset (green circles).

In addition to the comparison of the PDF we tested the DC method by means of a Perfect Sibling evaluation (PS)^15,66^. Since no observations of future climate exist, we used one GCM as pseudo-observations, to then try to predict the future evolution of that simulation using another independent simulation (Fig. 4). We selected the GFDL-ESM2M as the reference simulation (the ‘perfect sibling’ or ‘truth’) and compared with the same data from other 5 GCMs in the periods 1960–1990 and 2040–2069. *Raw* refers to the uncorrected data and DC after applying the bias-correction approach. We assessed the skill using the RMSE between the perfect sibling and the other GCMs. For this example, we selected South America, but we performed the evaluation for other regions and combinations of GCMs (Fig. 5).

Both for the example simulation and for all possible combinations, the RMSE decreases significantly applying DC compared with the uncalibrated case. For the South America region the error fluctuates between *E* = *246–387 mm season*^*−1*^ in the raw case and *E* = *122–159* *mm season*^*−1*^ (i.e. roughly 50% lower) in the DC case for DJF seasonal precipitation, and between *E* = *2–3.5* *°C season*^*−1*^ in the raw case and *E* = *0.4–1.4 °C season*^*−1*^ in the DC case for DJF seasonal mean temperature (Fig. 4). The correlation between corrected GCMs and the perfect sibling, considering all the 2,400 possible combinations (among GCMs, seasons, regions), is above 0.8 in virtually all cases (i.e. seasons, regions and GCMs) for both precipitation and temperature (Fig. 5, left). The RMSE for the same combinations is less than 200 mm season^−1^ (1.5 °C) for more than 90% (75%) of the cases for precipitation (temperature) (Fig. 5, right).

Overall, the evaluation suggests that DC produces reliable and robust future projections of the means of climate variables for use in impact assessment. However, we note that while mean seasonal conditions play a significant role in the eco-physiology of crops and wild species^45,67^, there are other aspects of climate projections that influence crops and biodiversity including the frequency and intensity of drought and/or hot spells, or the occurrence 1-day extreme precipitation events^15,68,69^. Some of these aspects, however, are not adequately simulated by GCMs^70,71^. More work is thus required to develop better models that can accurately simulate these events, or that generate plausible scenarios that can then be used into agricultural and species models. Additionally, other methods of bias-correction and downscaling exist (e.g. refs. ^25,30,37^) and can correct errors in the temporal aspects of GCM simulations. Future studies and datasets may focus exploring and comparing additional methods to DC or different implementations of DC (e.g. using different interpolation methods), especially as CMIP6 model outputs become available to the public, as different methods can produce varying results and thus add to the ‘uncertainty cascade’ in impacts modeling^16,72^.

## Usage Notes

### Recommendations to users

The World Data Center for Climate (WDCC) portal provides the open access high-resolution climate data presented in this article, with an associated permanent DOI (10.26050/WDCC/CCAFS-CMIP5_downscaling)^41^. In addition, the CCAFS-Climate portal (www.ccafs-climate.org) also provides the data, and includes useful explanations and documentation to help users understand the technical principles of the downscaling techniques and other useful information about the data. It also includes a *Contact Us* section providing user support via e-mail and an *About Us* section with institutional information and a quick guide to citing the data in peer-reviewed publications.

We provide data at four different resolutions (30 arc-s, 2.5 arc-min, 5 arc-min, and 10 arc-min), and encourage users of these data to understand the assumptions we made in producing them. The data provided here are intended to assess the impacts of changes in the mean climate state, especially as it relates to temperatures and precipitation, and the derived bioclimatic indices. For applications concerning changes in weather characteristics, extreme events, or interannual variability, users should find other datasets or bias-correction methods that address such aspects. As a whole, our assumptions might lead to uncertainties, and therefore, we suggest that users of these data perform a detailed uncertainty analysis in order to determine if these data in fact fulfil their requirements. We caution users regarding the uncertainties involved in our processes, and in no case should users understand these projections as future predictions of climate for particular places. Rather, the data should be understood as high-resolution and bias-corrected future projections in which a compromise is made between climate model physics and scale of analysis. It is noteworthy that as progress continues in climate modelling in the next decades, we expect that downscaling and bias-correction may no longer be required for using climate model output to assess the impacts of climate change.

Processing and storage capacity in research centers making use of these datasets might also be a limiting factor when using these data. We therefore suggest research centers to download the appropriate resolution datasets that suit their studies. We note that significant differences are of course present between 30 arc-s and 10 arc-min spatial resolutions. The former is the original WorldClim resolution, providing considerable detail on climatic patterns according to orography, whilst the latter, retrieves a credible high-resolution dataset, but with less level of detail.

### Applications in agro-environmental research

From 2014 to date, CMIP5 DC downscaled data from the CCAFS-Climate portal have been downloaded by nearly 1,400 users in more than 186 countries around the world. Approximately 394,000 data files have been downloaded, amounting to 119 TB of data. Users of the data include representatives from national government research institutions and the NGO sector as well as the research community. Moreover, to date more than 300 journal papers, 10 book chapters, and 40 theses or reports have cited the data shown here. These data have been used for a wide variety of purposes; some examples are summarized below.

Guo *et al*.^73^ used DC data to investigate the current status and distribution of the Quiver tree *Aloe dichotoma* in southern Africa and assess the projected future changes of its habitat under different climatic scenarios. The tree provides moisture to a wide variety of mammals, birds and insects, and its conservation is critical to maintaining the local ecosystem in the future. Jennings and Harris^74^ used DC data to identify specific climate and vegetation parameters for anticipating how, where and when ecosystem vegetation may transform with climate change across the southwestern USA. CCAFS-Climate portal data were used in conjunction with a weather generator to map environmental suitability for the Zika virus, showing that over 2.17 billion people in the tropics and sub-tropics live in areas suitable for the virus and its vector^75^.

The CCAFS-Climate portal data have been widely used to help identify analogue sites. Comparing present-day farming systems with their future analogues can facilitate the exchange of knowledge between farmers in different locations who share common climate interests and allows adaptation strategies and technologies to be tested and validated^76^. Over the last several years, more than 15,000 farmers have been testing new seed varieties in seven districts of India as a component of improved local seed systems, by selecting and testing varieties identified using climate analogue analysis, thereby enhancing smallholders’ resilience to climate change^77^. In another example, the International NGO Concern Worldwide is using analogue analysis to identify adaptation options and investment strategies in Chad and South Sudan^77^.

Many studies have used the CCAFS-Climate portal data to project the impacts of climate change on agricultural production. Several examples are included in working papers for crop production^78^, and for livestock production^79^, both focused on sub-Saharan Africa. In Timor Leste, government response to the 2016 El Niño included committing US$12 million to buy reserve food stocks, partly as a result of the use of CCAFS-Climate data^77^. Other study^80^ assessed the impact of global warming on outdoor ice-skating in Canada, and showed that its availability and benefits are projected to continue declining at an accelerated rate, posing a real challenge to this popular cultural ecosystem service.

CMIP5 DC data has also contributed as a main input of agriculture sector-specific studies performed in Africa. It includes an analysis of the impacts of climate change on cocoa in Ghana and Cote d’Ivoire^81^, the climate change impacts and potential benefits of drought and heat tolerance in chickpea in East Africa^82^, simulate impacts of climate change on water use and yield of irrigated sugarcane in South Africa^83^, potential benefits of drought and heat tolerance in groundnut for adaptation to climate change in West Africa^84^, analysis and mapping of climate change risk and vulnerability in Central Rift Valley of Ethiopia^85^, study of matching seeds to needs-female farmers adapt to a changing climate in Ethiopia^86^, among others.

A recent outcome assessment based on Outcome Harvesting^87^ shows that the data in the CCAFS-Climate portal are not only widely used in research activities but are also effective in contributing to development outcomes. The climate data are influencing, directly or indirectly, a range of societal actors, including funders investing in further research, NGOs and government agencies changing their programming and planning for climate change adaptation, and farmers and communities adopting new agricultural practices. The CCAFS-Climate portal is providing scientific, robust and credible climate information, but there are also secondary functions that are contributing to the achievement of outcomes, such as supporting visualization and communication about future climates, enhancing reflective and independent thinking, and engaging partners and stakeholders in collaborative activities^77^.

## Data Availability

The DC code used to produce the global database of future climates is publicly available under a Creative Commons Attribution 4.0 International license (CC BY 4.0). We carried out the procedure mainly in ArcInfo Workstation 10, and the R language for statistical computing^88^. The source code consists of two Arc Macro Language (AML)^89^ (version 10.0) and two R (version 3.2.4) programming language scripts^90^.
